# Supplementary material for: Fabrication of Laser-Induced Graphene Based Flexible Sensors Using 355 nm Ultraviolet Laser and Their Application in Human–Computer Interaction System
Source: Materials (Basel). 2023 Oct 29;16(21):6938. doi: 10.3390/ma16216938 (PMC10648489; doi:10.3390/ma16216938)
Supplement: Supplementary file 1 [file materials-16-06938-s001.zip › Supporting Information.pdf]

Supporting Information

Fabrication of Laser-Induced Graphene Based Flexible Sensors Using 355 nm Ultraviolet Laser and Their Application in Human–Computer Interaction System

Binghua Sun [1,2,3], Qixun Zhang [1,2,3], Xin Liu [1,2,3,*], You Zhai [1,2,3], Chenchen Gao [1,2,3] and Zhongyuan Zhang [4]

[1] Key Laboratory of CNC Equipment Reliability, Ministry of Education, School of Mechanical and Aerospace Engineering, Jilin University, Changchun 130025, China
[2] Chongqing Research Institute, Jilin University, Chongqing 401100, China
[3] Institute of Structured and Architected Materials, Liaoning Academy of Materials, Shenyang 110167, China
[4] College of Automotive Engineering, Jilin University, Changchun 130025, China
[*] Correspondence: liu_xin2006@jlu.edu.cn

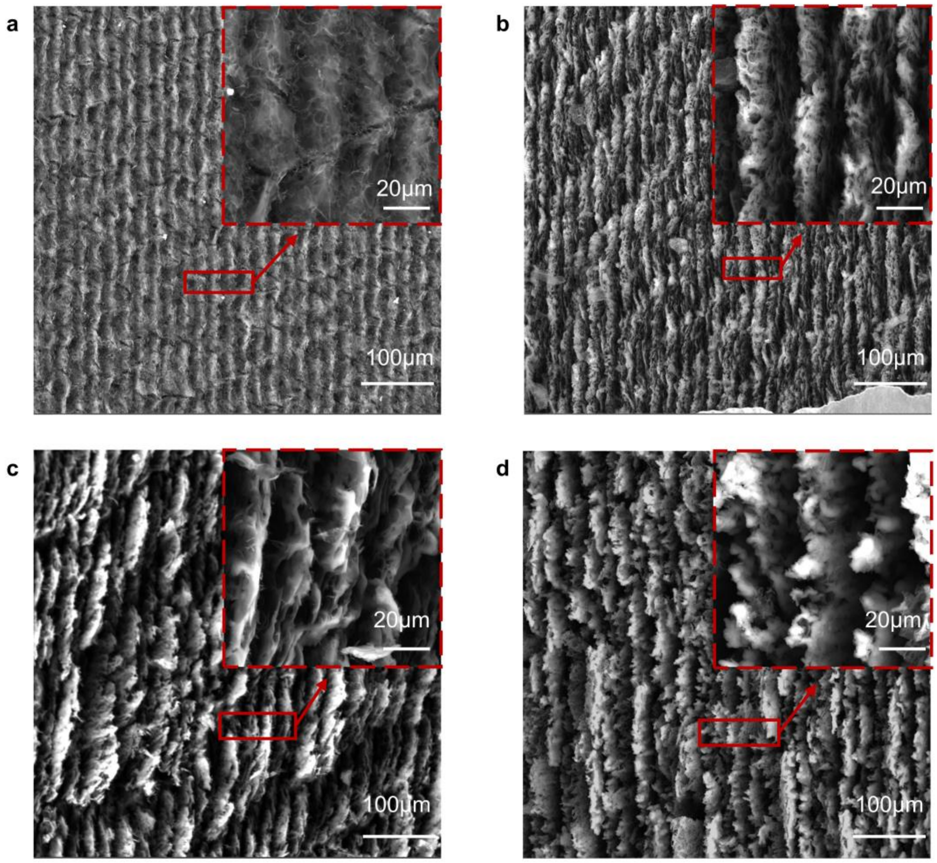

Figure S1: 1000× and 6000× SEM surface morphology of LIG prepared with different laser powers with unchanged scanning speed of 50 mm/s, processing numbers of 10 and scanning interval of 0.02 mm. a. 0.3 W. b. 0.6 W. c. 0.9 W. d. 1.2 W

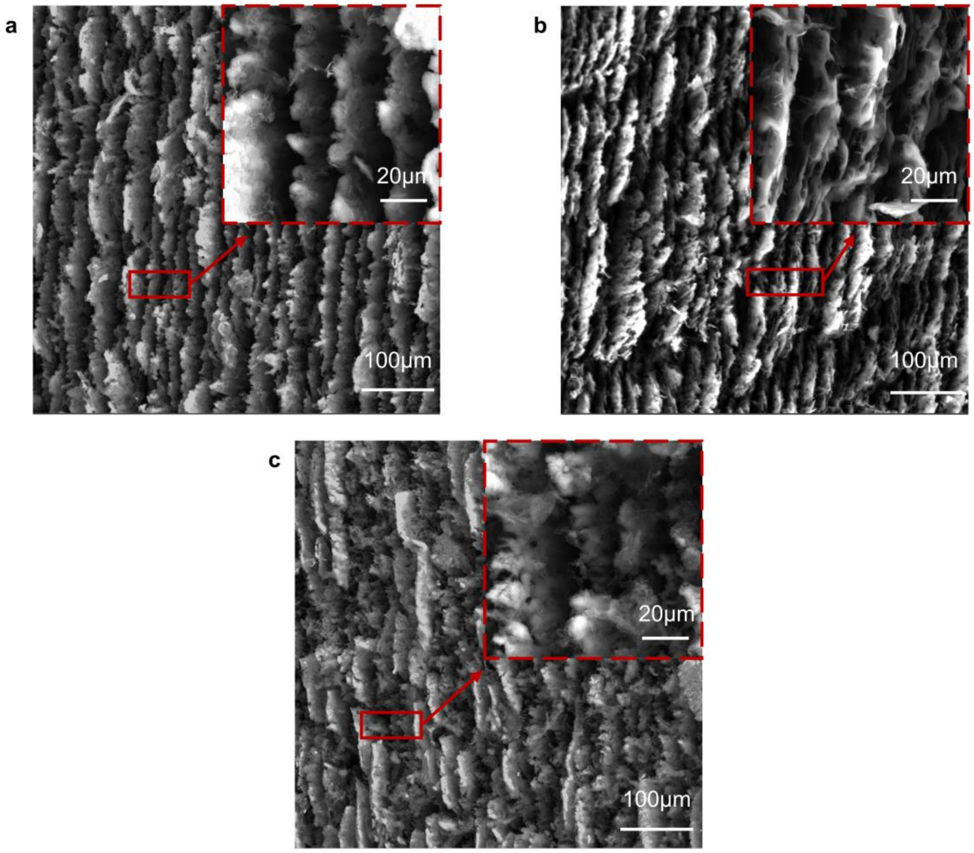

Figure S2: 1000× and 6000× SEM surface morphology of LIG prepared with different scanning speeds with unchanged laser power of 0.9 W, processing numbers of 10 and scanning interval of 0.02 mm. a. 30 mm/s. b. 50 mm/s. c. 70mm/s.

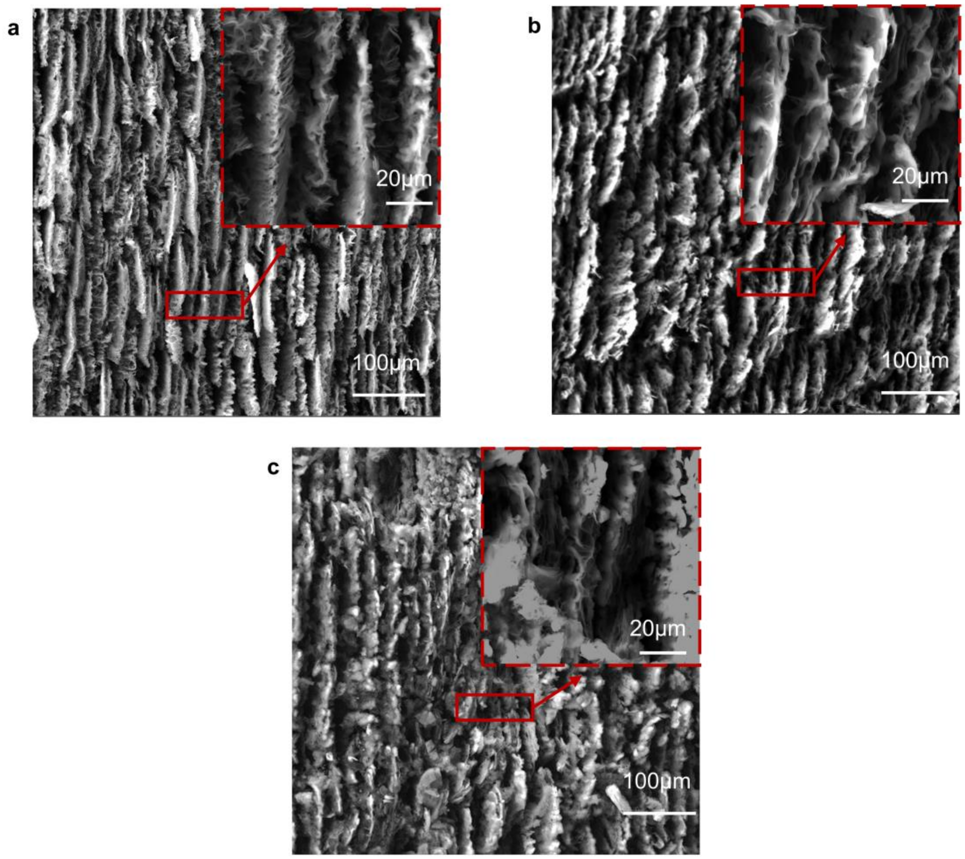

Figure S3: 1000× and 6000× SEM surface morphology of LIG prepared with different processing numbers with unchanged laser power of 0.9 W, scanning speed of 50 mm/s and scanning interval of 0.02 mm. a. 5. b. 10. c. 15.

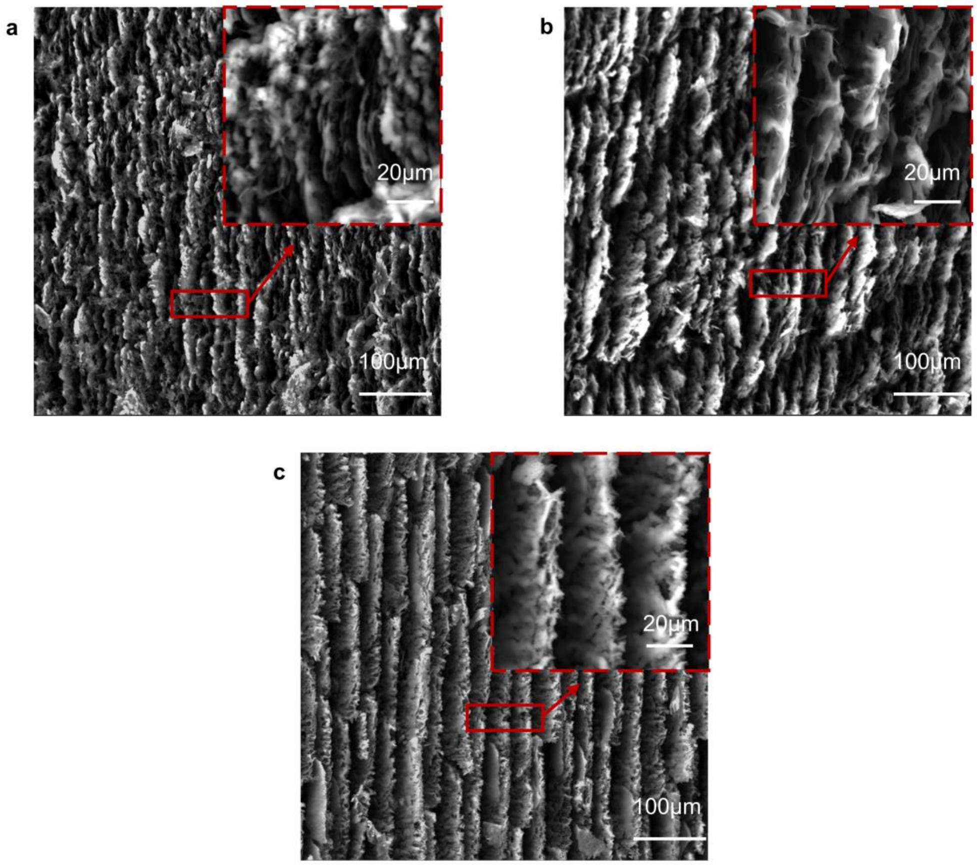

Figure S4: 1000× and 6000× SEM surface morphology of LIG prepared with different scanning intervals with unchanged laser power of 0.9 W, scanning speed of 50mm/s and processing numbers of 10. a. 0.01 mm. b. 0.02mm. c. 0.03mm.
